# *ALGR*: A multi-purpose agricultural landscape generator in R

**DOI:** 10.1371/journal.pone.0334745

**Published:** 2025-10-30

**Authors:** Eyal Goldstein, Antonia Deutscher, Eamon O’Keeffe, Kerstin Wiegand

**Affiliations:** 1 University of Göttingen, Ecosystem Modelling, Göttingen, Germany; 2 Present address: University of Kassel, Grassland Science and Renewable Plant Resources, Kassel, Germany; 3 University of Göttingen, Centre of Biodiversity and Sustainable Land Use (CBL), Göttingen, Germany; University of Ferrara, ITALY

## Abstract

Agricultural and ecological modelers commonly use maps as input for spatially explicit simulations. While real world maps are often used, they are limited by being static objects, therefore making it difficult to assess how patterns within the landscape contribute to ecological processes. Agricultural landscape generators (ALG) are a useful tool for simulating maps in a more flexible way. They can increase robustness of models that rely on landscape maps as input, they allow modelers to give spatial representation to non-spatial models, and they are a useful tool for recreating spatial patterns in agricultural-dominated landscapes. A limitation of previous ALGs is that they have rarely been designed for general use (non-open source software, not written in R, and designed for specific projects). Furthermore, they are typically either extremely general and thus oversimplified or have a high specificity for particular use cases. *ALGR* bridges this gap by providing a general-purpose, dynamic landscape generator that balances structural realism with adaptability. *ALGR* generates agricultural landscapes with a three-step approach: first, outlining potential space, second, field placement inside of that space, and third, enrichment of the landscape with information. This stepwise approach ensures that *ALGR* generates landscapes with realistic spatial patterns while remaining adaptable to diverse regions and applications. It is the first ALG that is specifically designed to allow a simple integration within the R programming environment and the *r-spatial* package environment. *ALGR* is designed as a general-purpose generator, which is simple to use and facilitates an easy integration in modelling workflow. We present several examples of workflows using *ALGR*, to demonstrate its usefulness. Our examples include: 1) simulating different land use shares, 2) parameter tuning of *ALGR* to recreate real world landscape, patterns 3) spatially distributing crop portfolios, and 4) using real-world maps as a basis for field placement.

## Introduction

Landscape maps are fundamental to agricultural and ecological modeling, providing the spatial structure necessary for simulating ecological and economic processes. Use of landscape maps can be found in agent-based models [1–3], mechanistic models [4–6], as well as different bio-economic models [7,8].

While these maps enhance model realism by grounding simulations in actual landscapes, they also introduce several challenges that limit their broader applicability. Maps of real-world landscapes are static objects, often making it difficult to assess how different spatial distributions and patterns within the landscape contribute to the process of interest. Additionally, even if the pattern-process relationships can be disentangled, the unique characteristics of a landscape map taken from a specific location makes the transferability of insight to other locations a difficult matter [9].

To enhance the generalizability of model insights and disentangle the effects of spatial patterns on agricultural and ecological processes, researchers have developed Agricultural Landscape Generators (ALGs), which generate synthetic maps that mimic real-world landscape mosaics and serve as flexible model inputs [9]. These ALGs can generally be divided into two categories: pattern-based generators and process-based generators. Pattern-based generators are more simplistic and take an algorithmic approach to create geometric properties. In contrast, process-based generators aim at creating landscape patterns by modelling the underlying socio-economic, ecological, and topographic mechanisms and processes that have created current landscape patterns.

Despite their potential, ALGs have remained underutilized due to barriers such as limited accessibility, lack of integration into widely used programming environments, and the inherent tradeoff between generality and realism. The majority of ALGs have not been written in R, the programming language used most by ecologists [10]. Landscape generators built outside of R can hinder reproducibility and complicate integration with common ecological analysis workflows, limiting their accessibility and practical utility for many researchers. Additionally, ALGs often struggle to balance generality and realism. Many ALGs show high generality and transferability, but they often produce oversimplified landscapes. This is particularly true for many pattern-based generators. For example, Inkoom et al., 2017 [11] developed a neutral agricultural landscape generator using a simple tessellation algorithm that can be used anywhere in the world, but lacks realism. Conversely, there are highly specialized ALGs which can simulate landscapes in great detail, but these cannot be easily generalized or transferred to other locations outside of the region for which they were developed. This mostly applies to process-based generators. For example, Salecker et al., 2019 [12] developed a process based agricultural landscape generator that uses a combination of social and geographical parameters from the Jambi region of Indonesia. While the work by Salecker et al., 2019 [12] offers a high level of realism, it is not easily transferable to other locations. While some work has been done towards creating ALGs that maintain high generality while also maintaining a high degree of realism [13,14], they still lack an easy pathway for integration in the workflow of the wider modelling community.

To bridge the gap between realism and generalizability, we introduce *ALGR*, a multi-purpose and dynamic landscape generator that combines pattern-based and process-based approaches. It is specifically designed for seamless integration within the R programming environment, ensuring accessibility and flexibility for researchers. Our approach follows a three-step procedure. First, a potential space map is generated, delineating areas available for field placement based on simulated landscape features such as slope, soil, or agricultural constraints. Second, a pattern-based approach is used to place fields within this space. Finally, the landscape is enriched with additional information, including farmer identities and crop distributions. The three-step simulation maintains structural realism, i.e., maps correspond with their real-world counterparts in terms of landscape configuration and geographic properties. This is achieved by mimicking some of the processes of real-world landscape generation [15].

This approach is suitable for many regions where landscape composition and configuration are strongly influenced by natural features, such as topography and soil. This applies to diverse regions worldwide, including China [16], the United States [17], Australia [18], and Europe [19]. It is important to note that landscapes where configuration is primarily shaped by external processes, such as road-driven deforestation or traditional social structures, are better modelled using a purely process-based artificial landcover generator such as EFForTS-LGraf [12]. Our novel approach allows for a wider use of ALGs in scientific research. To increase its flexibility, our landscape generator includes several methods for generating potential spaces, as well as different algorithms for field placement. *ALGR* also allows for adding further information to the landscape such as crops, field IDs and farmer information, making it easily integrable with complex ecological models. To increase the accessibility of this landscape generator, we have also encapsulated the code in R package format, which is easily accessible to ecologists interested in using *ALGR* for their own projects.

### The *ALGR* package

**Fig 1:**
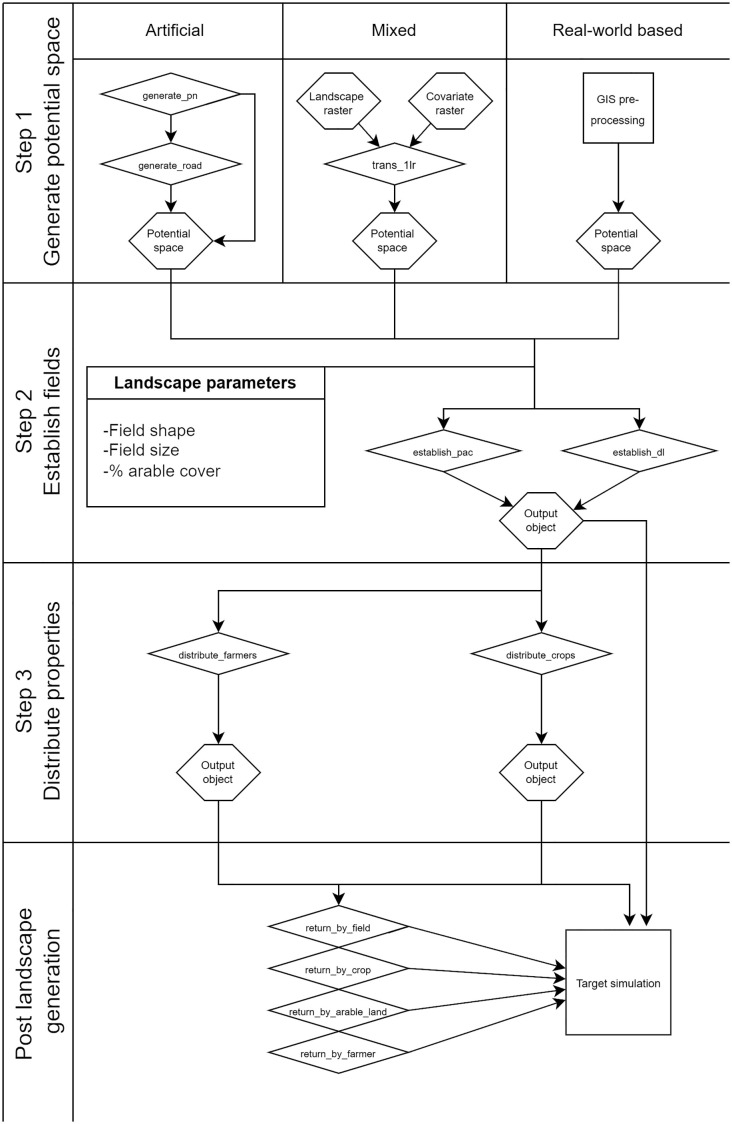
A flowchart illustrating the step-by-step functions of landscape generation: generating potential space, establishing fields inside of the potential space, and distributing properties to the fields. Hexagons: data objects, rhombuses (diamonds): *ALGR* functions, squares: external functions. Additionally, the package includes post-generation functions that enable plotting and extracting various types of raster data from the *ALGR* output object.

**Fig 2:**
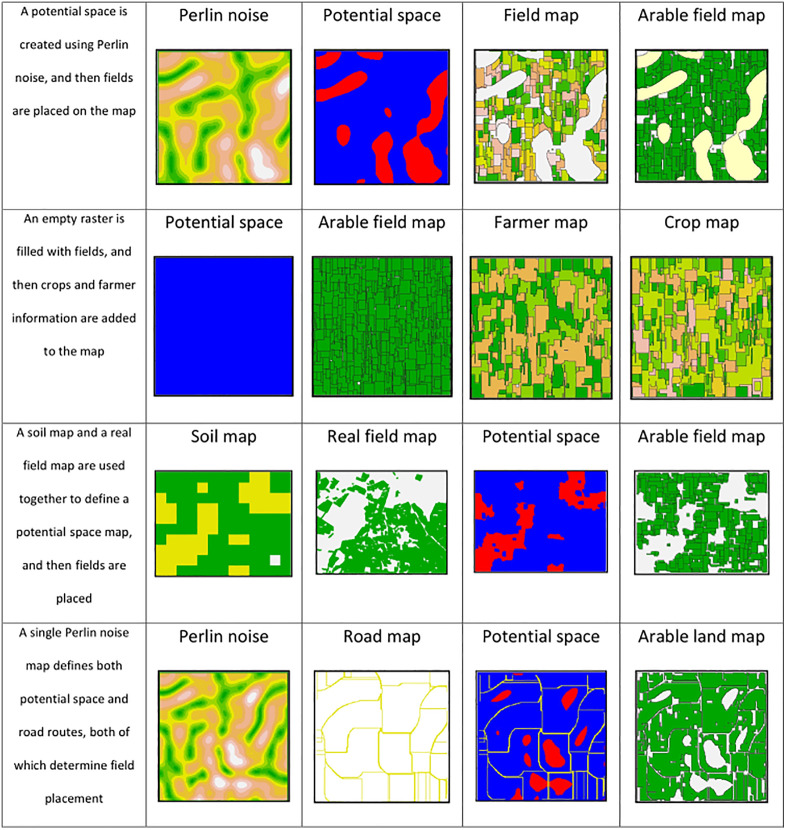
A visual demonstration of the pathways used to generate different types of maps. For each of the potential space maps blue represents available space, while red represents cells that are not part of the potential space. Different output maps are possible, including arable field maps, arable land maps (excluding field borders), crop maps, and farmer maps.

*ALGR* generates agricultural landscapes with a three-step approach: first, outlining potential space, second, field placement inside of the potential space, and third, enrichment of spatial information. The functions belonging to the different steps can be seen in Fig 1, and the example maps resulting from the different functions can be seen in Fig 2.

We designed the landscape generator using a stepwise, modular approach for several key reasons. First, modularity provides flexibility, allowing users to adapt the workflow to diverse use cases. For instance, if the default Perlin noise-based method for generating potential space is unsuitable, users can easily substitute it with an external map. Second, users can engage only with the steps relevant to their needs—those uninterested in crop placement or farm ownership, for example, can stop after generating a map of arable land. Finally, this modular structure facilitates the integration of individual components into other workflows or models.

### First step: outlining potential space

Three functions are included in the *ALGR* package to outline potential agricultural space. The potential space for arable land can be generated using two built-in methods, Perlin noise or the hybrid world approach. Alternatively, an external raster map with designated arable areas can be imported for use as potential space.

### Perlin noise approach

The Perlin noise algorithm is an image generator based on the creation of a controlled stochastic effect [20]. It has been widely used for animation [21] and simulation of artificial natural patterns, including terrain [13,22]. *ALGR* uses a Perlin noise algorithm to stochastically generate a potential space map that simulates terrain-driven agricultural limitation. The flexibility of the Perlin noise algorithm allowed us to also simulate patterns seen in small-scale soil distribution, coarse soil characteristics (referred to as *aglim* in FAO soil categorization), and rough terrain features such as slope and elevation.

The ‘*generate_pn’* function in *ALGR* is a wrapper around the ‘*noise_perlin’* function from the *ambient* package, which is then adapted for specific use. The function takes the raster generated by the ‘*noise_perlin’* function and converts it to a map with values ranging from 0-90^o^. This map can then be used in three different ways: 1) it can be categorized into potential space based on a map value threshold specified in the parameters of ‘*generate_pn’*, 2) it can be categorized based on a defined share of potential space cover specified in the parameters of ‘*generate_pn’*, 3) or it can be left as raw data for further manipulations before being used as input for the second step of the landscape generator.

### Hybrid world approach

The hybrid world approach models artificial agricultural landscapes within the context of existing terrain. Where a field can be placed (i.e., the potential space) depends on landscape characteristics such as slope, soil, and/or *aglim*. The ‘*trans_1lr*’ function helps determine which areas on the map are likely to be suitable for agriculture. It calculates the probability of each cell in the raster being part of the potential agricultural space, based on two types of input: categorical land cover maps with a single ‘arable landcover’ category and covariate maps. After probabilistically assigning the potential space cells, the function converts neighboring cells by a majority vote to simulate spatial autocorrelation. In other words, the function ensures that areas designated as potential space are clustered together rather than scattered randomly, which results in a potential space area that is larger than just arable land on the original land cover map.

The idea behind this approach is to facilitate the simulation of an arable land configuration in a real-world context. The potential space created using this method contains not only the current location of arable fields, but also possible locations where fields could be placed but are not. Additionally, these maps contain valuable information such as potential space located on suboptimal topography or soil.

### Additional artificial input: roads and rivers

The package functionality also supports the use of additional raster layers as input, which can further refine the potential space and exclude field placement. This functionality is generally reserved for spatial features such as roads and rivers, allowing them to be included when they are of interest for the output of the generator. We have also provided a tool for artificially generating roads (‘*generate_road’*) and rivers (‘*generate_river’*) using a path of least cost algorithm based on slope maps (either real world slope maps or artificially generated Perlin noise maps).

### Second step: field placement

Second, *ALGR* employs a pattern-based approach to place fields inside the potential space. The package currently includes two different methods for field placements, a place-and-conquer algorithm, and a dead-leaves algorithm. Both algorithms share a set of parameters for field establishment, including: field shape, field size, distribution of size (normal or log-normal), and the percentage of potential space to be covered by fields. The difference between the two approaches is that the place-and-conquer algorithm is slower but generates more realistic results, while the dead-leaves algorithm is quicker but the landscapes are less realistic. Benchmarks for speed of landscape generation for landscapes of different sizes are listed in supporting information figure S6 in S1 Appendix.

### Place and conquer algorithm

‘*establish_pac’* uses an algorithm that places each field by segments. The algorithm starts with drawing a field shape value (relation between width and length) and field size value from a distribution defined in the parameters. These values define the length of each segment, and the number of segments to be placed during the establishment of a given field. Each segment is aligned as closely as possible to the previous one, while maximizing both the alignment between both segments and preventing each new segment from being placed on forbidden cells (i.e., outside of the potential space or on another field). This process continues until the field either reaches its pre-set size (all segments have been placed) or there is no more potential space available.

The main advantage of this approach is that it generates very realistic landscapes, where the shape and size of each field is a result of both the parameters and the constraints of the space. The main disadvantage of this method is that it is slightly slower than the dead leaves algorithm for very large landscapes.

### Dead leaves algorithm

‘*establish_dl*’ is based on a modified dead leaves algorithm. In this approach, the potential space is first completely filled using the traditional dead leaves algorithm, including the distribution of randomly drawn rectangular shapes and sizes to be placed inside of the potential space. The modified algorithm then includes a second step where the algorithm starts removing fields beginning from the smallest one, until the percentage of potential space to be covered by fields is reached. The removal of the smallest patches (rather than random patches) is done to avoid the over-fragmentation of the landscape, due to unrealistically many small fields.

The main advantage of this algorithm is its speed. A very large landscape can be generated quickly, but with a cost to realism of the field shape, size, and placement.

### Third step: post-hoc farmer and crop distribution

The *ALGR* package allows for enriching the spatial information by adding crop types and farmer information to the different maps, as well as plotting-tools.

The general structure of the output data from all field establishment functions includes a list of individual fields, with each field containing slots for the following information: field location, field identity, farmer identity, and crop identity. This structure is used to add information to the fields through designated functions but can also be accessed and changed directly.

Farmer identity can be assigned using the function ‘*distribute_farmers’*. The parameters used for this are the mean and standard deviation of the number of fields assigned per farmer and the distribution type (uniform, normal, log normal). The fields are assigned to one farmer at a time until all fields are attributed to farmers. This means that the number of farmers in a landscape is decided by the distribution of fields per farmer and the total number of fields in the landscape, rather than directly parametrized. The fields can be either assigned in a spatially structured way, where nearby fields are given to the same farmer, or in a random way, where the farmer gets random fields from the entire landscape. Additionally, farmers can be directly mapped to individual fields using the function ‘*assign_farmers_by_table’*.

Crop allocation to fields is done post-establishment using the ‘*distribute_crops’* function. This function takes a list of crops, and the percentage of arable land they should occupy in the landscape, and then distributes them between the fields. Because only a single crop is assigned to each entire field, larger generated landscapes or those with smaller fields will have better correspondence between the desired cover percentages and the resulting cover percentages in the landscape. Additionally, crops can be directly mapped to individual fields using the function ‘*assign_crops_by_table’*.

### Testing and validation

We randomly selected 30 different 2 km × 2 km landscapes from Lower Saxony as reference landscapes for calculating landscape metrics used in our systematic testing. Using the *landscapemetrics* R package [23], we calculated a set of metrics for each landscape, including number of patches, patch area (mean and standard deviation), largest patch index, contagion index, edge density, Euclidean nearest neighbor (mean and standard deviation), patch shape index, landscape shape index, and fractal dimension. To identify *ALGR* parameter settings that generated landscapes matching specific metric values, we employed a genetic algorithm where each chromosome represented a parameter combination, and fitness was defined by how closely the generated landscape’s metrics matched those of the reference. After obtaining the top parameterization for each landscape-metric combination, we generated 100 landscapes per setting—totalling 131 700, and computed the different metrics values. We then calculated a final score by quantifying the scaled distance between generated and reference metrics, and used 1 minus the final score for analysis, where 1 indicates a perfect match and 0 a complete mismatch. The different tests performed on the landscape generator can be seen in figures S1-S5 in the supporting information in S1 Appendix). Additionally, we included an assessment of *ALGR*’s scaling up which can be seen in figures S6-S7 in the supporting information in S1 Appendix).

## Example applications

We illustrate the potential of *ALGR* with four examples. The description and results of two examples are included in the main text and the others are in the supplementary information. For each of the examples we included an R notebook in the supplementary information for reproducing the example using the *ALGR* package.

### Example 1: Land use share scenario

Here, we generate landscapes with varying shares of potential space. This potential space is then allocated between arable fields and seminatural habitat, with both the share and spatial configuration of fields determined by the parameters of the field establishment function. Using landscape metrics, we analyze how changes in the share of potential space and arable fields influence the characteristics of seminatural habitat patches.

In agricultural landscapes, nature conservation policies often focus on conservation by increasing the share of seminatural habitats [24]. An example is the goal of the EU common agricultural policy to maintain 10% of all agricultural area as seminatural habitat [25]. These seminatural patches can vary in several properties that affect biodiversity and ecosystem services across the landscape, including location within the landscape, size of patches, as well as connectivity and distance between patches of seminatural habitat.

Models dealing with biodiversity and ecosystem services in agricultural landscapes often use real-world maps as an input for simulations (e.g., Holland et al., 2016 [24]; Kirchweger et al., 2020 [7]). The downside of this approach is that these real-world maps are static objects, making it hard to disentangle the effects of seminatural cover from spatial configuration.

In this example, we show how to use *ALGR* to create a scenario where share of both arable land and seminatural habitat vary within the landscape according to a gradient, along with variation in spatial configuration. Configuration changes arise from two sources: adjustments to field shape and size parameters, and the stochastic effects of different initialization seeds. Starting with a Perlin noise simulation of the topography, we continuously expand the potential space that is available for arable field placement, using ‘*generate_pn’*. This potential space is then divided between arable fields and seminatural habitat in varying shares using ‘*establish_pac’*.

**Fig 3:**
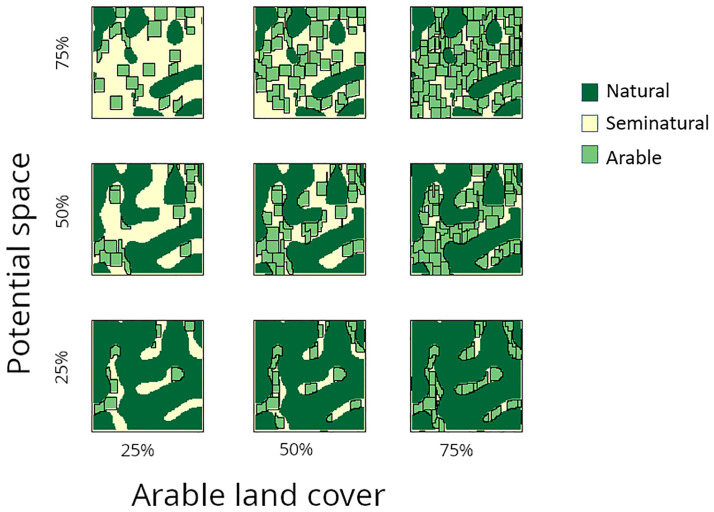
A visualization of the analysis design for example 1, land use share scenario. Landscapes differ along the y axis in the percentage of the map available as potential space and along the x axis in the percentage of the potential space occupied by arable land cover.

In this example, the parameters were distributed using a Latin hypercube design, with equal intervals of 1% for the potential space (25–95%), intervals of 1% for the part of the potential space to be covered by arable land (25–95%), as well as 4 field sizes (0.5–2.5 ha and intervals of 0.5 ha), and 4 field shapes which define the ratio between width and length of fields (1–3 and intervals of 0.66) (Fig 3).

Finally, we profile the spatial configuration of seminatural habitat patches across the different landscapes using the *landscapemetrics* R package [23]. For the spatial configuration profile we use a set of metrices: *mean patch area*, *largest patch index*, *fractal dimension*, *perimeter area ratio*, *Euclidian nearest neighbor distance*, and *number of patches*.

**Fig 4:**
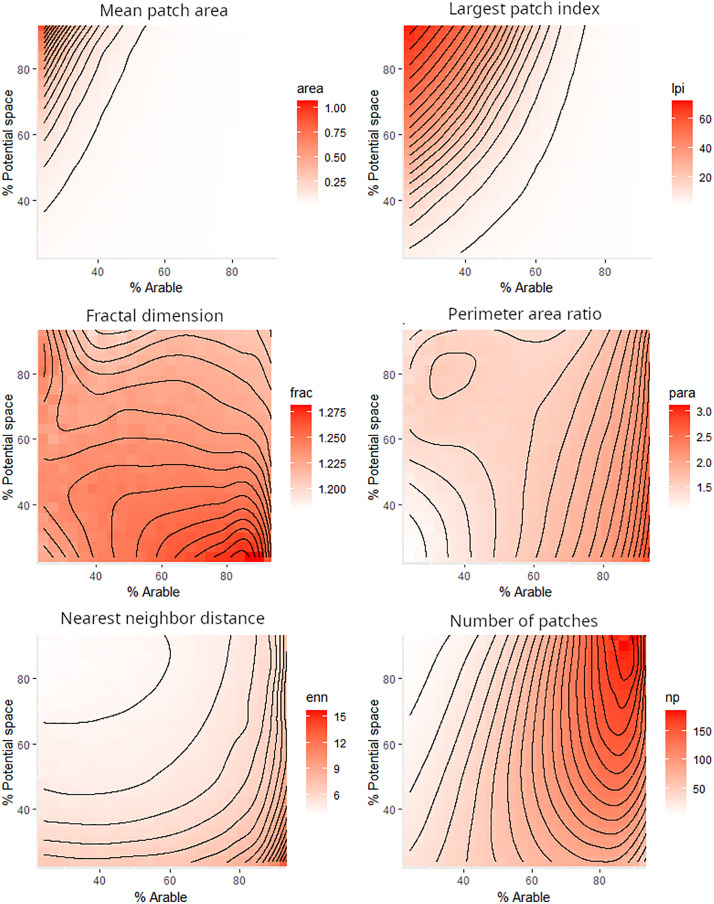
The landscape metrics profile of the seminatural habitat patches across a double gradient where both the potential space and the arable cover vary. For definitions of metrics see Hesselbarth et al. (2019).

For the size-related metrics *mean patch area* and *largest patch index* (Fig 4), the largest patches of seminatural habitat were obtained when percentages of potential space and seminatural habitat were the highest (top left section of the plot). For the shape related metrics, *fractal dimension* and *perimeter-area-ratio*, the signal was less clear. *Fractal dimension* of seminatural habitat patches was highest for low percentages of potential space seminatural habitat (bottom right of plot), i.e., when seminatural patches were smallest (see size-related metrics). However, the distribution across the gradient did not show a clear pattern. The *perimeter-area ratio* of seminatural habitat patches was mostly driven by the percentage of arable cover, with the highest perimeter per area at high arable cover, regardless of the percentage potential space (right side of the plot). The *Euclidean nearest neighbor distance* showed a pattern similar to the size-related metrics, where the smallest mean *Euclidean nearest neighbor distance* between patches of seminatural habitat occurred when percentages of potential space and seminatural cover were highest (top left section of plot). For the number of patches, we found a maximum where the number of seminatural patches was highest when both potential space and arable cover were approximately 90%. Comparable results were achieved for patches of agricultural land (see Fig S8 in section ‘Example 1 – Land share scenario: further results’ of supporting information in S1 Appendix).

Most importantly, our results show that that none of the metrics are linear in their response to changes in the share of potential space or arable cover in a landscape. The same change in share of potential space or arable cover will have a different effect on patch structure and configuration, which is often driven by landscape characteristics such as topography. These results are particularly relevant for agricultural policies that promote an increase in seminatural landcover across a range of agricultural landscapes and can be used by modelers to demonstrate differential effects of specific policies.

### Example 2: Pattern reconstruction of agricultural landscapes using a genetic algorithm

Here, we demonstrate how to use a genetic algorithm [26] to fine-tune the parameters of the artificial landscape generator. This process generates maps that share specific characteristics (landscape metrics) with real-world maps.

Landscapes can be characterized by multiple metrics, with each one conveying a certain aspect of the spatial composition and configuration of a landscape [27]. The spatial features represented by these metrics affect ecological processes, for example fragmentation facilitates metacommunity structure [28], landscape diversity and simplification can affect ecosystem functions such as pollination or pest control [29], and structural connectivity within the landscape can facilitate animal movement [30].

Because real landscapes cannot be freely manipulated, it is difficult to disentangle which element of a landscape facilitates a specific ecosystem function of interest. With a simulation approach we can create landscapes with predefined properties and assess their ability to support ecological processes. Here we show how to use a genetic algorithm to parameterize the *ALGR* landscape generator with the goal of recreating the spatial characteristics of a real-world landscape. More specifically, the goal is to keep certain metrics of the landscape fixed, while allowing for variation in other metrics and the overall arrangement. Using the *ALGR* in this way can help modelers generate a diversity of maps while controlling for specific properties of the landscape in each map.

**Fig 5:**
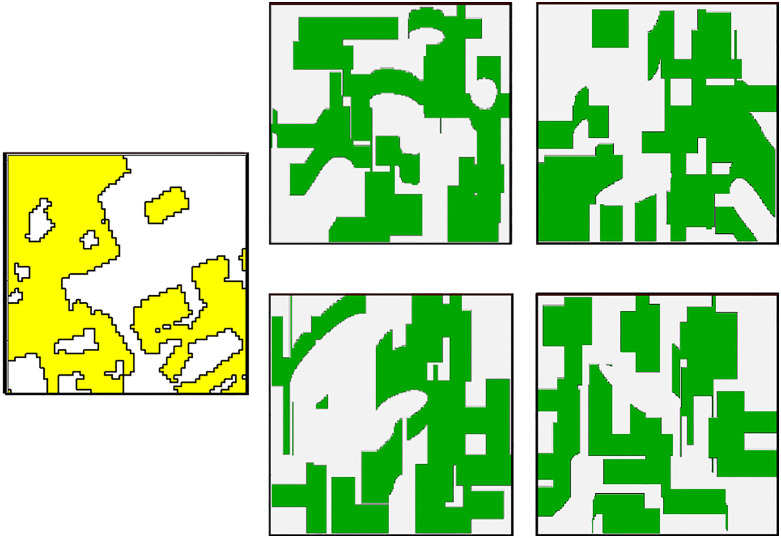
A reconstruction of the landscape pattern of a 2 km × 2 km landscape using the metrics *number of patches* and mean *patch area*. Yellow: reference landscape used as optimization goal for our genetic algorithm. Green: four realizations obtained using the optimized *ALGR* parameters.

**Fig 6:**
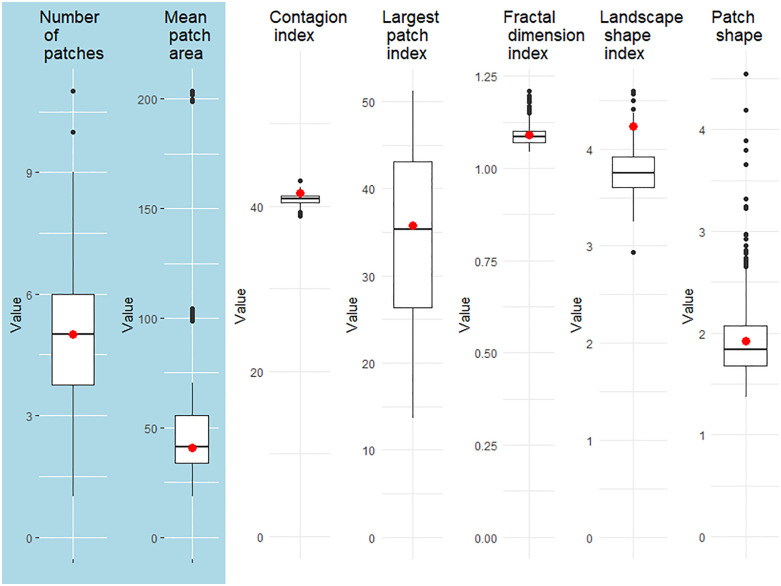
A comparison of different landscape metrics between our 300 generated maps (boxplots) and the reference landscape (red dots). Blue background: metrices that were used as optimization goals in our genetic algorithm, including *number of patches* and *mean patch area* (ha), and white background: additional metrices that were not part of the optimization.

In our example, we used a 2 km × 2 km map as reference. We then used a set of landscape metrics to quantify the optimization goal in our genetic algorithm: the *number of patches* and *patch area* (mean and standard deviation). These two metrics are commonly used in spatial characterization of landscapes. Using the metrics of our reference map as optimization goals, the genetic algorithm searched for an optimal parameterization of *ALGR*. Here, optimal means that *ALGR* generates landscapes that have maximal similarity to the original reference landscape in terms of metric values. Once a parametrization was found (note that several solutions are possible), we can generate any number of maps that are equivalent to the reference map, i.e., similar or even identical in terms of our chosen landscape metrics but different in spatial arrangement (see Fig 5). We generated 300 maps using the optimal parameterization from the genetic algorithm and compared them to our original reference maps using a list of additional landscape metrics that were not used for the genetic algorithm optimization, including: *contagion index* (measuring how likely a grid cell of one category will have a neighbor cells of the same category), *largest patch index*, *fractal dimension*, *landscape shape index,* and *patch shape index* (see Fig 6). Our genetic algorithm converged within 30 generations (see Fig S10 in section ‘Example 2 – Pattern reconstruction of agricultural landscapes using a genetic algorithm’ of supporting information in S1 Appendix).

### Example 3: Crop portfolio to landscape scenario

This example demonstrates how non-spatial crop share portfolios are translated into spatially explicit, artificially generated landscapes. We then use simple landscape metrics to demonstrate how these different realizations might affect a spatially explicit ecological process (see section ‘Example 3: Land use allocation problem’ in supporting information in S1 Appendix).

### Example 4: Real world maps as potential space

This example includes the use of real-world maps for the potential space and demonstrates how to generate fields on top of the potential space (see section ‘Example 4: using real-world maps as a basis for landscape simulation’ in supporting information in S1 Appendix).

### Example 5: Monte Carlo uncertainty propagation

This example uses a Monte Carlo uncertainty propagation approach in order to measure the error in the model outputs under chosen parametrization (see section ‘Example5: measuring error uncertainty propagation’ in supporting information in S1 Appendix).

## Conclusion and outlook

*ALGR* is the first agricultural landscape generator readily available within the framework of R that allows for systematic and reproducible landscape generation. It can be used to simulate maps needed for agricultural and ecological models that require landcover maps as input, allowing for generality of use across different contexts and regions while maintaining structural realism. While other ALGs have been previously published [9], *ALGR* has the advantage of being available in open-source format and well-integrated in the R workflow, the most prevalent programming environment used by ecologists.

The *ALGR* package is specifically designed to facilitate the different needs of ecologists and modelers interested in using an ALG in their workflow. Besides the multiple functionalities presented in this paper, the package is designed to always output an object that stores both field information as well as farmer information, therefore allowing the maps to be easily integrated with more sophisticated ecological and socio-economic models, as well as be further manipulated using simple R code.

Though *ALGR* is general in its application, it was developed with European agricultural landscapes in mind. In these regions, the configuration of agricultural landscapes is often determined by the availability of ‘suitable’ space for arable land. Thus, in its current version, other factors are not included which are of higher importance in other regions, such as proximity to water bodies (e.g., North Africa) or roads (e.g., Indonesia, Brazil). While *ALGR* includes multiple algorithms capable of generating complex landscapes, they still constitute a simplification of the real world. The Perlin noise algorithm is capable of generating hierarchical neutral landscapes [31], and has been previously used as part of the process for generating agricultural landscapes [12]. Despite that, soil and slope are shaped by historical geological processes which would not be easily captured by a Perlin noise algorithm. Perlin noise can introduce spatial biases when applied in regions with high geological complexity. By design, Perlin noise produces smoothly varying gradients, which may be less suitable for modelling high geological complexity. In mountainous terrain, for instance, steep slopes and shallow soils can sharply delimit arable land, both of which would not be easily captured by Perlin noise. Applying Perlin noise in such settings may therefore lead to unrealistic placement of arable fields and underrepresentation of sharp ecological constraints, potentially biasing subsequent ecological or socio-economic analyses. For this reason, we emphasize the importance of selecting algorithms that are appropriate for the landscape context. *ALGR* offers two alternatives and allows users to either choose a mixed-world approach, which delineates potential space according to different covariates, or incorporate external potential space maps to overcome these limitations. Additionally, it offers only a simplified version of many factors that determine the landscapes. For example, the enrichment stage relies on simplified algorithms that may not fully capture real-world patterns that are influenced by socio-economic and historical factors. For instance, fields can be assigned to farmers either based on proximity to other fields already assigned to the same farmer or entirely at random. When these two default options fail to reflect the desired distribution, users can directly access the *ALGR* output object and assign fields to farmers using custom code. Overall, it’s important to properly choose the right method for generating a landscape according to the specific needs of the study.

While *ALGR* is capable of simulating real-world landscapes with high accuracy, in order to fully understand how stochastic variation in landscape configuration affects agricultural, ecological, and economic dynamics, then *ALGR* needs to be coupled with further models. For the purpose of analyzing ecological dynamics in agricultural landscapes, there are many models which simulate animal movement and habitat use (e.g., Gardner et al., 2024 [32]; Häussler et al., 2017 [5]; Pe’er et al., 2011 [33]). For simulating more complex socio-economic dynamics, there are many other models which can be integrated with *ALGR,* including agent-based models that simulate the complex sociological and economic behaviors of individual actors or agents. Many such models have already been developed for European agricultural systems (reviewed in Huber et al., 2018 [34]). For example, AgriPolis was developed to simulate agent decision in land markets and product markets in the EU [35], FOM was designed to simulate crop allocation and farm profit [36], and CRAFTY can be used to simulate non-homogenous agent behavior under different scenarios and societal pathways [37], just to mention a few. These models offer potential frameworks for bio-economic modelling along with *ALGR*, allowing for a more complex approach to socio-economic dynamics in agricultural landscapes. Finally, real-world applications often involve incomplete or uncertain data. To address this, we recommend conducting a sensitivity analysis by running the model multiple times across a plausible range of parameter values.

In comparison to earlier ALGs reviewed by Langhammer et al. (2019), *ALGR* is distinguished by its ease of parameterization and balanced design. Many previous ALGs required complex parameterization, which limited their usability and hindered their adoption in broader modeling workflows [38]. Others were either too abstract to capture realistic spatial patterns [39] or too narrowly tailored to specific case studies [12], limiting their transferability across regions. *ALGR* addresses these limitations by combining a modular architecture with simple but flexible parameter controls, enabling users to define key characteristics such as field size, shape, spatial constraints, and land-use proportions. This allows researchers to generate structurally realistic but highly configurable landscape mosaics, suitable for exploring landscape-level questions across ecological, economic, and land-use domains. Despite these advantages, *ALGR* cannot handle all natural irregularities, and is dependent on algorithmically generating spatial patterns which can lead to over organized landscapes. Researchers requiring high levels of realism should evaluate if *ALGR* can reproduce the desired patterns for their study.

To facilitate the use of the package, we have included a notebook to reproduce each one of the examples presented in this paper. The help sections also include working examples of all functions. Finally, we have also included a vignette that provides a full description of each one of the functions included in the package which is also hosted on the same github repository.

## Supporting information

S1 Appendix Supplementary information on paper. (DOCX)

S1 File Code examples. (ZIP)
