# Supplementary material for: ALGR: A multi-purpose agricultural landscape generator in R
Source: PLoS One. 2025 Oct 30;20(10):e0334745. doi: 10.1371/journal.pone.0334745 (PMC12574932; doi:10.1371/journal.pone.0334745)
Supplement: S1 Appendix — (DOCX) [file pone.0334745.s001.docx]

**Supplementary information on paper, ALGR: A multi-purpose agricultural landscape generator in R**

**Validation of ALGR:**

**Reference maps**

As a set of reference landscapes from which we extract all the landscape metrics used for our systematic test, we randomly choose 30 different 2 km × 2 km landscapes from lower saxony (Figure S1). The original maps were taken from the lower saxony. The landscapes were first converted to raster with resolution of 1m X 1m, and then aggregated to a resolution of 10 m × 10 m, in order to make sure the landscape metrics could be calculated at a scale relevant for arable fields without being skewed by small landscape features. We then calculated for each one of the landscapes a set of metrics using the *landscapemetrics* R package (Hesselbarth et al., 2019). The metrics that we calculated were: *number of patches*, *patch area mean*, *patch area sd*, *largest patch index*, *contagion*, *edge density*, *Euclidean nearest neighbor mean*, *Euclidean nearest neighbor sd*, *patch shape*, *landscape shape*, and *fractal dimension*.

**Genetic algorithm**

In order to find the parameter settings in ALGR that best generated landscapes with a specific landscape metric or landscape metric combination value, we used a self-adaptive genetic algorithm. The general idea is that the genetic algorithm takes as its population a set of chromosomes, each one consisting of a specific parameter combination of the ALGR parameters. These parameter combinations are used to produce landscapes, and then the landscapes are measured for the same landscape metrics as the reference maps. The difference between the goal landscape metrics and the landscape metric value for the generated landscape are then used to define the fitness of each chromosome.

**Testing the top chromosome**

After each genetic algorithm provided a top parametrization (chromosome) for every landscape and metric combination, we then generated 100 landscapes using those parametrizations, totaling in 131 700 landscapes. For each one of the generated landscapes we calculated its landscape metrics, and then measured the distance between those same metrics for in the reference landscapes to the ones in the generated landscapes, scaled using the minimum and maximum for these landscape metrics across all reference landscapes. Finally, for the sake of convenience we used the value of 1- final-score so that 1 is a perfect match and 0 is the lowest possible mismatch.

To evaluate how well the top parametrizations could reproduce the characteristics of the original landscapes, we conducted a series of analyses. In the first analysis, we selected the best-performing parametrizations for each individual metric and assessed how accurately the resulting landscapes replicated the values of those same metrics. We plotted the distribution of their final scores (Figure S2). In a second analysis, we extended this approach to two metrics: for each of the 30 landscapes, we evaluated how well the generated landscapes reproduced the values of both target metrics and visualized the results (Figure S3). Finally, we conducted a complementary analysis in which, instead of evaluating performance on the two metrics used to select the top parametrizations, we assessed how well the generated landscapes reproduced the remaining, non-target metrics collectively (S4-S5).

**
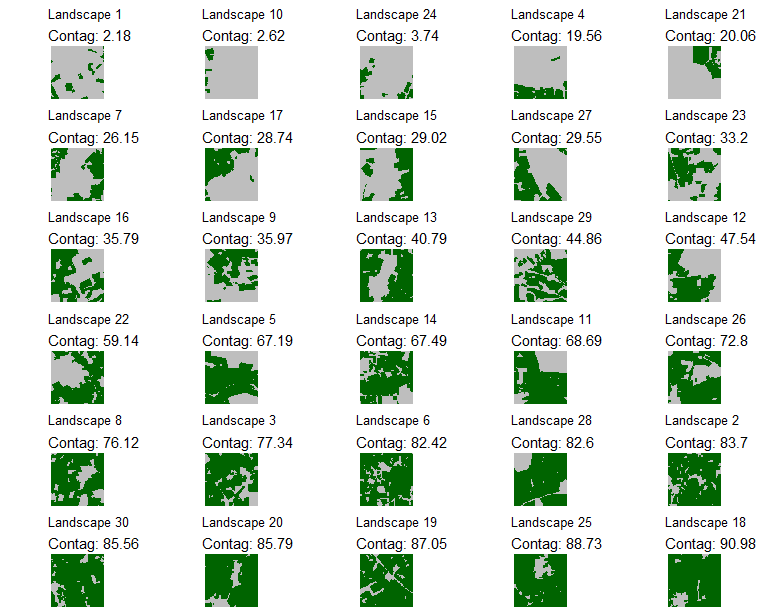
**

**Figure S1: Reference maps:** a set of 30 random landscapes taken from Lower Saxony and sorted according to the value of their contagion index (*Contag*.), ranging from 0-100. The landscapes represent a gradient in both configuration and composition, with varying degrees of arable cover represented in green.


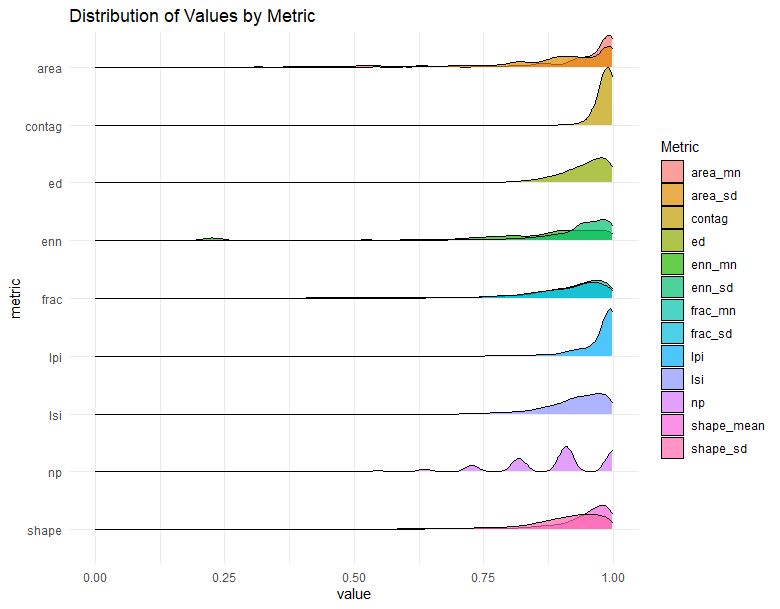


**Figure S2. Accuracy of top parametrizations for individual metrics.** For all landscape shown in Figure S1, we used a genetic algorithm to identify the top-performing chromosome—that is, the parametrization that produced landscapes most similar to the original in terms of a specific metric. We then ran each selected parametrization 100 times and plotted the distribution of the resulting scores. A score of 1 indicates a perfect match between the generated and original landscape for the metric of interest.


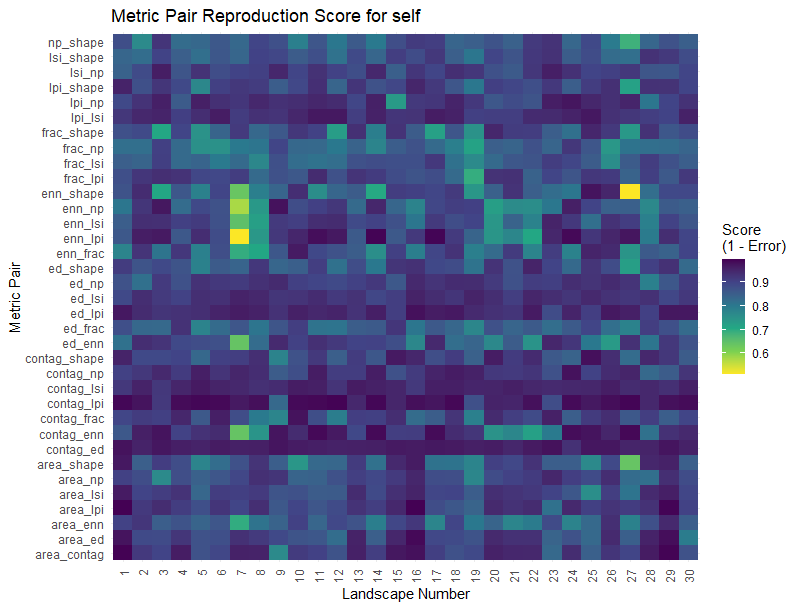


**Figure S3. Accuracy of top parametrizations across two target metrics.** For each landscape, we used a genetic algorithm to identify the parametrization that best reproduced the values of two selected metrics from the original landscape. This parametrization was then run 100 times, and we plotted the mean performance score for each landscape. A score of 1 indicates a perfect match between the generated and original landscapes with respect to the two target metrics.


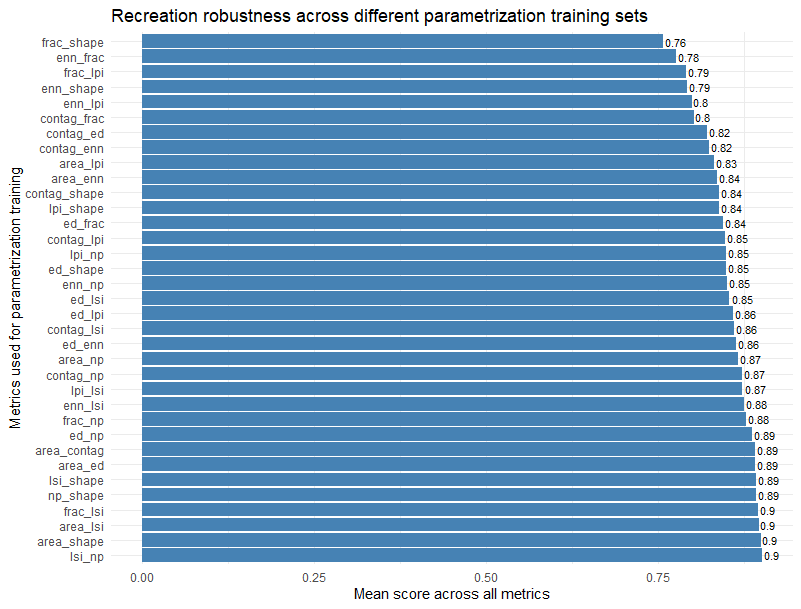


**Figure S4. Generalizability of top parametrizations beyond target metric pairs.**
For each landscape, we used the genetic algorithm to identify the parametrization that best reproduced the original values of a given pair of metrics. We then ran this parametrization 100 times and calculated the mean similarity across all 10 metrics, providing a measure of how well ALGR could reproduce non-target metrics after being tuned to just one metric pair.


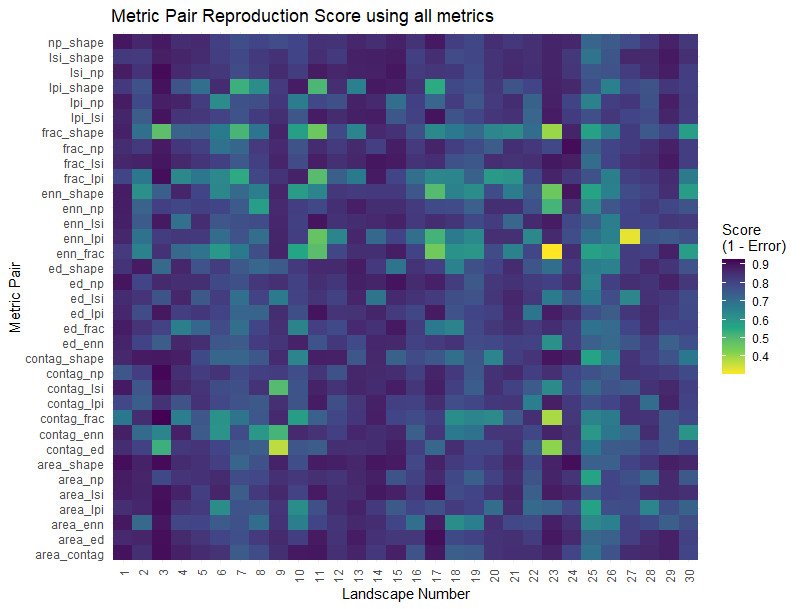


**Figure S5. Robustness of ALGR parametrizations across landscapes and metric pairs.** For each landscape, we used the genetic algorithm to identify the parametrization that best matched the original values of a given metric pair. We then ran each parametrization 100 times and calculated the average similarity across all 10 metrics. This analysis provides a measure of how well ALGR can generate landscapes resembling the original real-world landscapes, even when tuned using different metric pairs.

**Scaling of ALGR:**

**
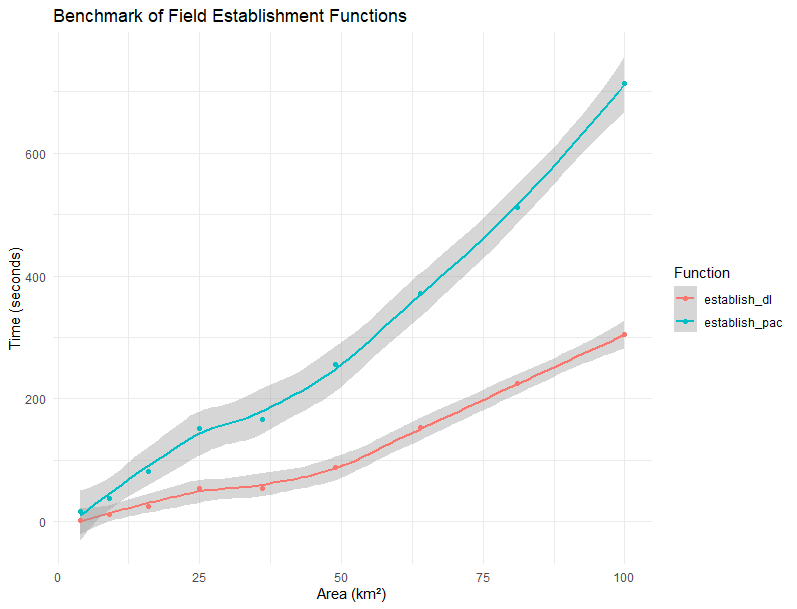
**

**Figure S6:** Scaling of ALGR

| A) | 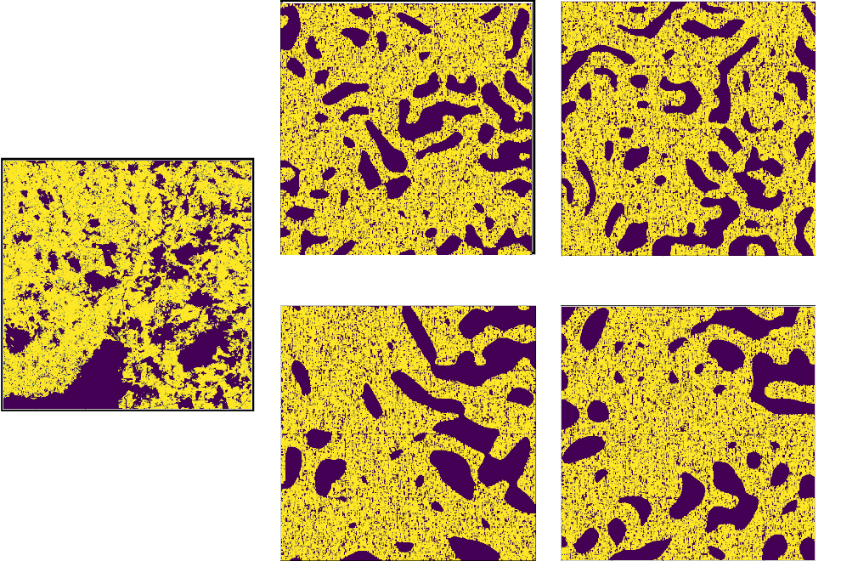 |
| --- | --- |
| B) | 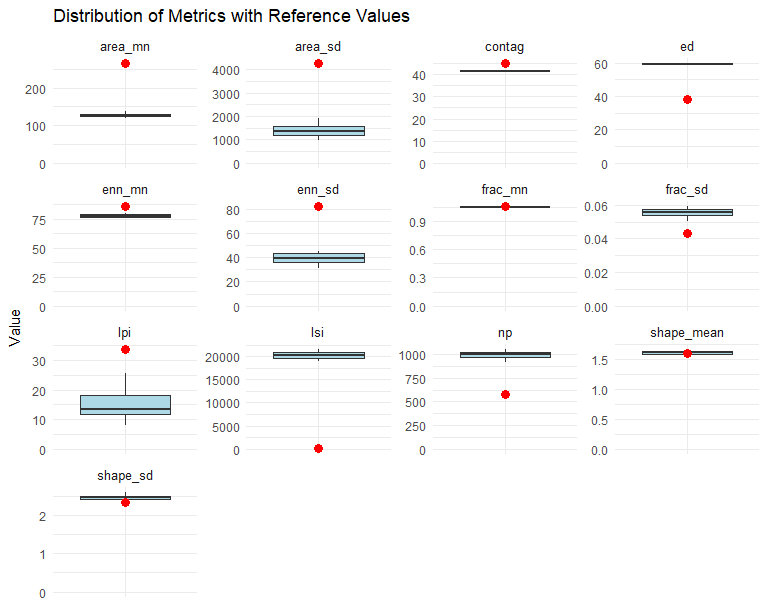 |

**Figure S7:** A reconstruction of the landscape pattern of a 50 km × 50 km landscape using the metrics *number of patches* and *patch area*. A) On the left is the original map and on the right are four reconstructed maps according to the top chromosome from the genetic algorithm. B) Metrics values from 30 simulated maps using the top chromosome. Red dots show the metric values of the original map in red dots. The reconstructed maps showed lower mean field size and higher number of patches in comparison to the original map, meaning that the reconstructed results consistently had a higher degree of fragmentation in comparison to the original.

**Example 1 – Land share scenario: further results**


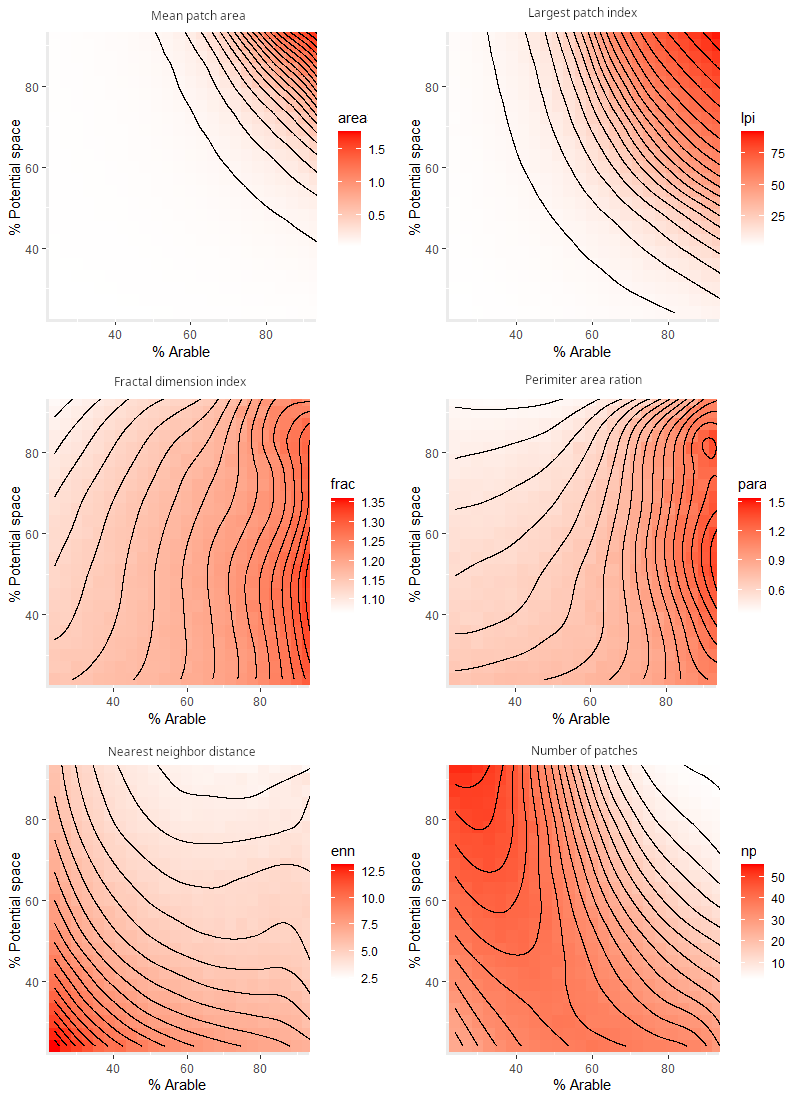


Fig S8: Landscape metrics profiles of the arable land patches across a gradient where both the potential space (% Potential space) and the arable cover (% Arable) vary between simulations.


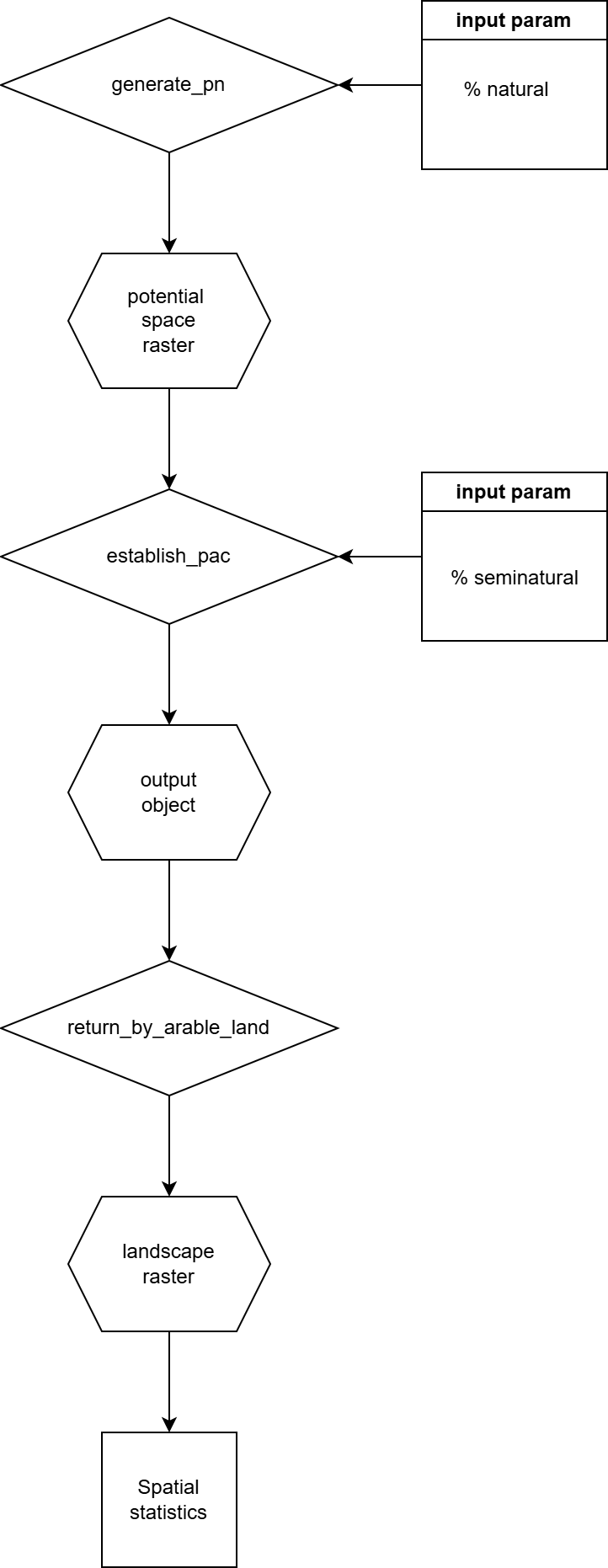


Fig S9: A flow chart of the different functions and objects used during example 1

**Example 2 – Pattern reconstruction of agricultural landscapes using a genetic algorithm** **: further results**

**
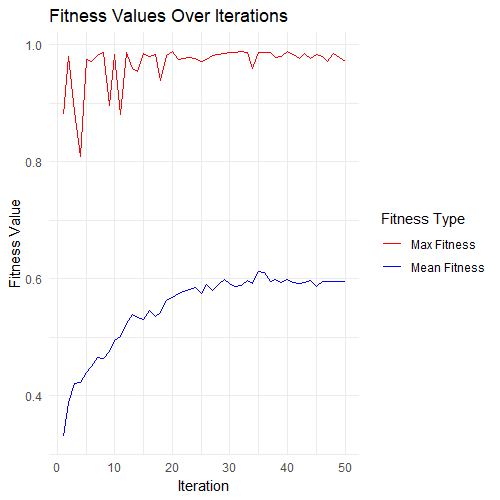
**

Fig S10: evaluation of the fitness function for our optimization of parameters in example 3A-B


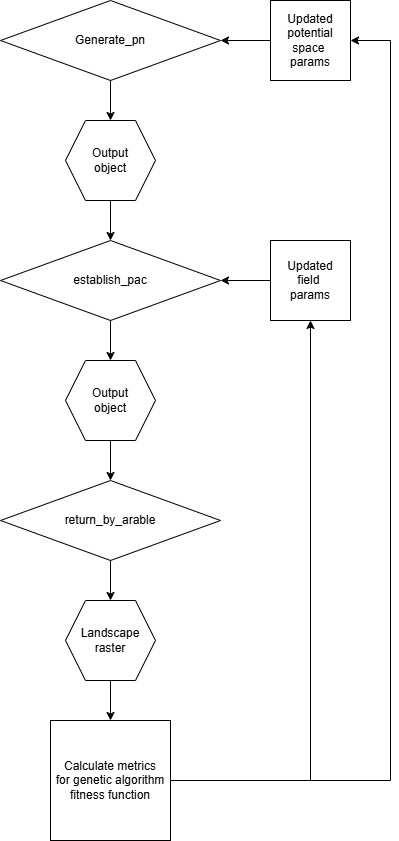


Fig S11: A flow chart of the different functions and objects used during example 2

**Example 3: Land use allocation problem**

Land use allocation according to different criteria is a known challenge in sustainable development (Kaim et al., 2018) and a general question for agricultural economists. Land use portfolio optimization, in other words finding the share of different land use categories which together best provision for different goals or purposes, is a common tool in land use multicriteria decision making. One major drawback for this method is the lack of consideration for space during optimization, as well as lack of spatial representation of its results. In ecology, in particular spatial representation is important because many ecological processes are spatially dependent (Hanski, 1998; Tscharntke et al., 2005), for example wild pollinators can use different land cover categories for feeding and nesting, and their ability to provide pollination services is influenced by the landscape structure (Tscharntke et al., 2012).

In this example, we show how to transform non-spatial land-use portfolios to spatially-explicit maps using ALGR. The portfolios that we used (Cong et al., 2014) were exclusively optimized for profit under different risk values. Risk is defined as the maximum accepted variance of profit return (see Fig 5 for portfolio composition). These portfolios were the so-called ‘statics’ portfolios in Cong et al. (2014) (see Fig 3 in Cong et al. 2014). The values were extracted using a graph digitization software (<https://automeris.io/>). We then generated landscapes using the ‘*establish_pac’* function, completely filling up the landscape with fields (100% potential space and 100% field placement). We then used the ‘*distribute_crops*’ function to allocate the crop types according to their share in the portfolio.

To analyze the implications of the different portfolios for pollination ecosystem services, we focused on the distance between feeding resource cells (rapeseed cells) and nesting resource cells (grassland cells). While rapeseed requires (wild or domesticated) bees for pollination, it does not provide good nesting resources for wild bees (citation). Grasslands, on the other hand, can provide a good nesting resource, but the rapeseed fields must be within a certain, species-dependent distance for bees to provide adequate pollination. To estimate levels of pollination, we calculated the mean distance to the nearest grassland cell for all rapeseed cells. For each portfolio, 5000 landscape realizations were generated across a gradient of varying field sizes. For each landscape, we calculated for each rapeseed cell the distance to the nearest grassland cell. We plotted the results as the mean across the mean-nearest-distance of all landscapes and estimated 95% confidence intervals using the lower 2.5% and upper 97.5% quantiles.

Our results show significant differences between portfolios in terms of pollination, as estimated for each landscape via the mean nearest distance to grassland cells for each rapeseed cell (See Fig S12C). There was a clear trade-off between portfolio risk and pollination. Portfolios calculated for high-risk high-return (3/4 Var) showed lower mean minimal distance to nearest grassland cells. This was mostly because grassland was a low-risk low-return land use category in the Cong et al. 2014 static model, which lead to a decrease in grassland as the risk level was increased. For the high-risk portfolios as well as for the observed portfolio, field size had a strong effect on the increase in mean minimal distance, highlighting the importance of multiple spatial consideration for a full assessment of land use composition effects on spatially explicit ecological processes. Our simulation results also demonstrate new levels of tradeoff beyond just risk and return, which can be calculated using landscape simulation methods.

**
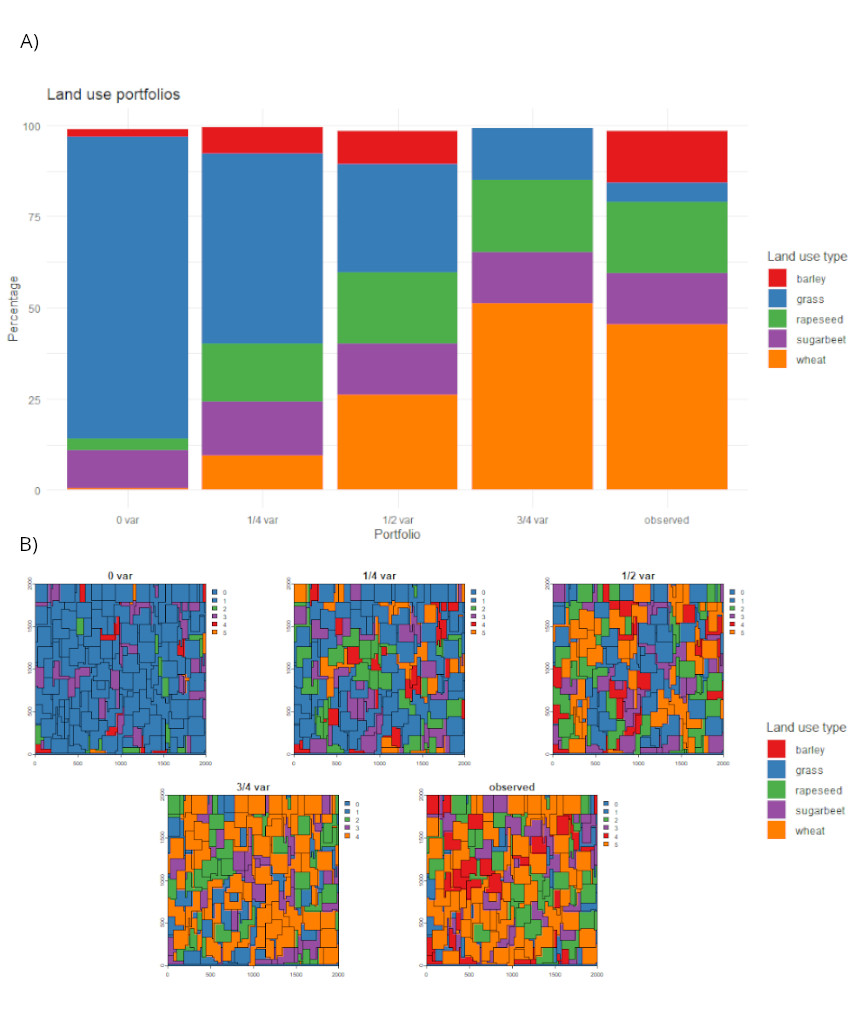
**


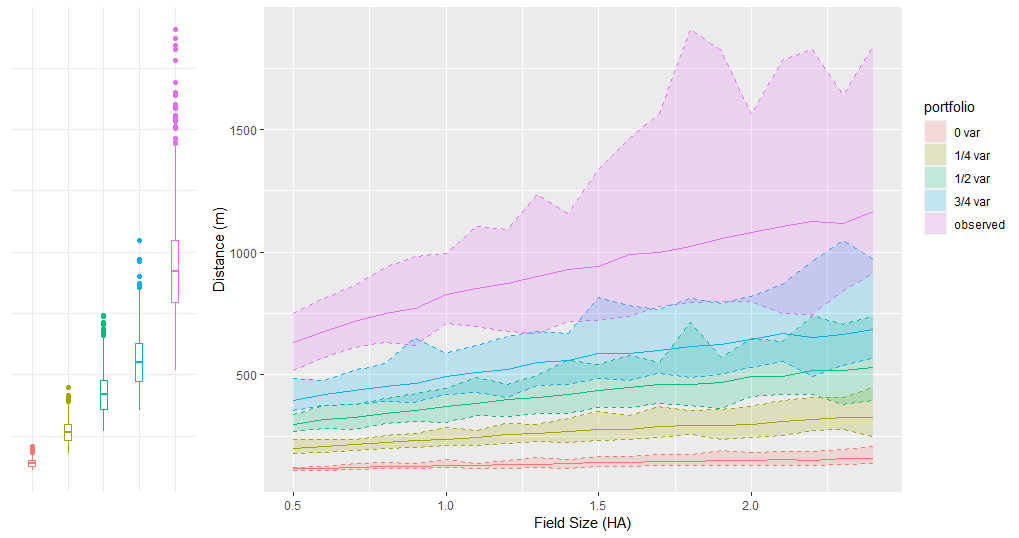


Fig S12: A) bar plot of each portfolio composition used in this example, B) one example of spatially explicit representation for each portfolio generated using the ALGR package and a field size of 0.5-3 ha, C) Mean distance between each rapeseed cell and the nearest grassland cell for the five portfolios (see legend). The boxplots lower hinge representing the 25^th^ percentile and the upper hinge representing the 74^th^ percentile, and the whiskers representing extend to 1.5 of the interquartile range. The continuous function (right) represents the mean distance (continues line) and the lower and upper bound of mean distance using the 1^st^ and 99^th^ percentile. The mean distance generally increases as the field size increases.


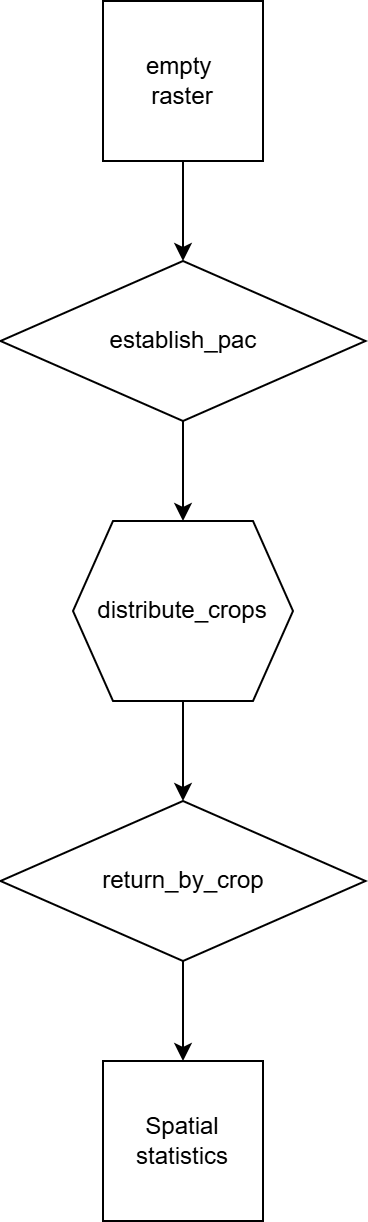


Fig S13: A flow chart of the different functions and objects used during example 3

**Example4: using real-world maps as basis for landscape simulation**

In the following example, we demonstrate how real world landcover maps can be used along with ALGR to simulate landscapes. For this example, we took a 12 km × 12 km section of the Copernicus EU landcover map from southern Germany that shows the dominant landcover category for each 100 m × 100 m raster cell. The maps that we used had three different landcover categories: forest, grassland, and arable land. In our example we considered all cells marked as arable land as potential space. When then took 2 km × 2 km sections of the map and downscaled them to a spatial resolution of 10 m × 10 m. Finally, we used the map sections as input for the *‘establish_pac’* function, which places fields across the potential space.

Our results demonstrate the possibility of using an external raster as input for ALGR (As shown in Fig S14A-C). This option can be particularly useful when there are certain unmovable categories (e.g. forest and grassland), but the cover and configuration of arable fields is changeable. Each one of the examples in S14C can be change in configuration by changing field sizes, shape, share of arable landcover, and change in initialization seed.


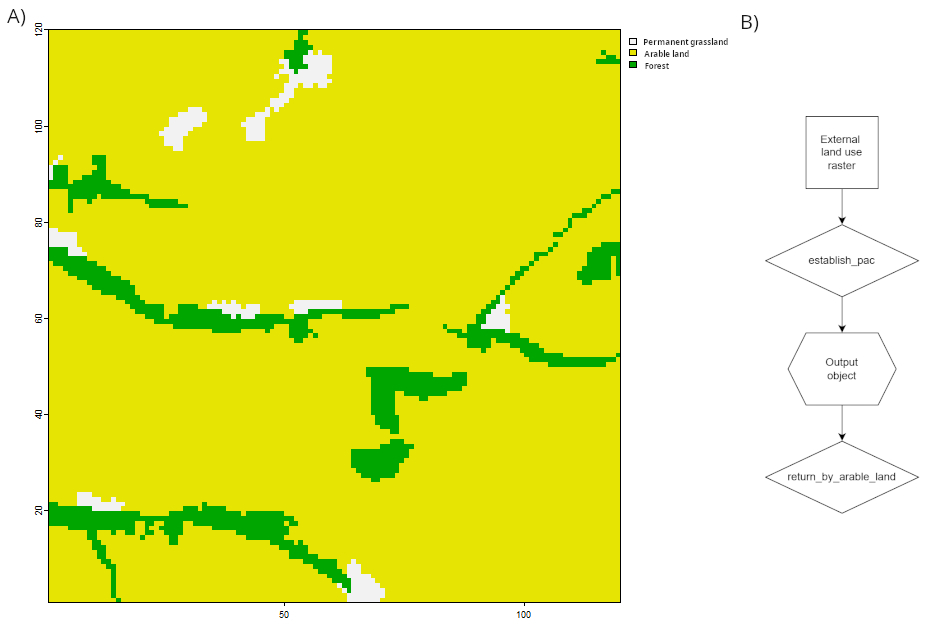


S14: A) A 12km x 12km map of dominant land use categories taken from the Copernicus landcover dataset. B) The flowchart for using an external map as input for ALGR. C) example of field placements on the ‘arable’ land use category of the Copernicus input map.


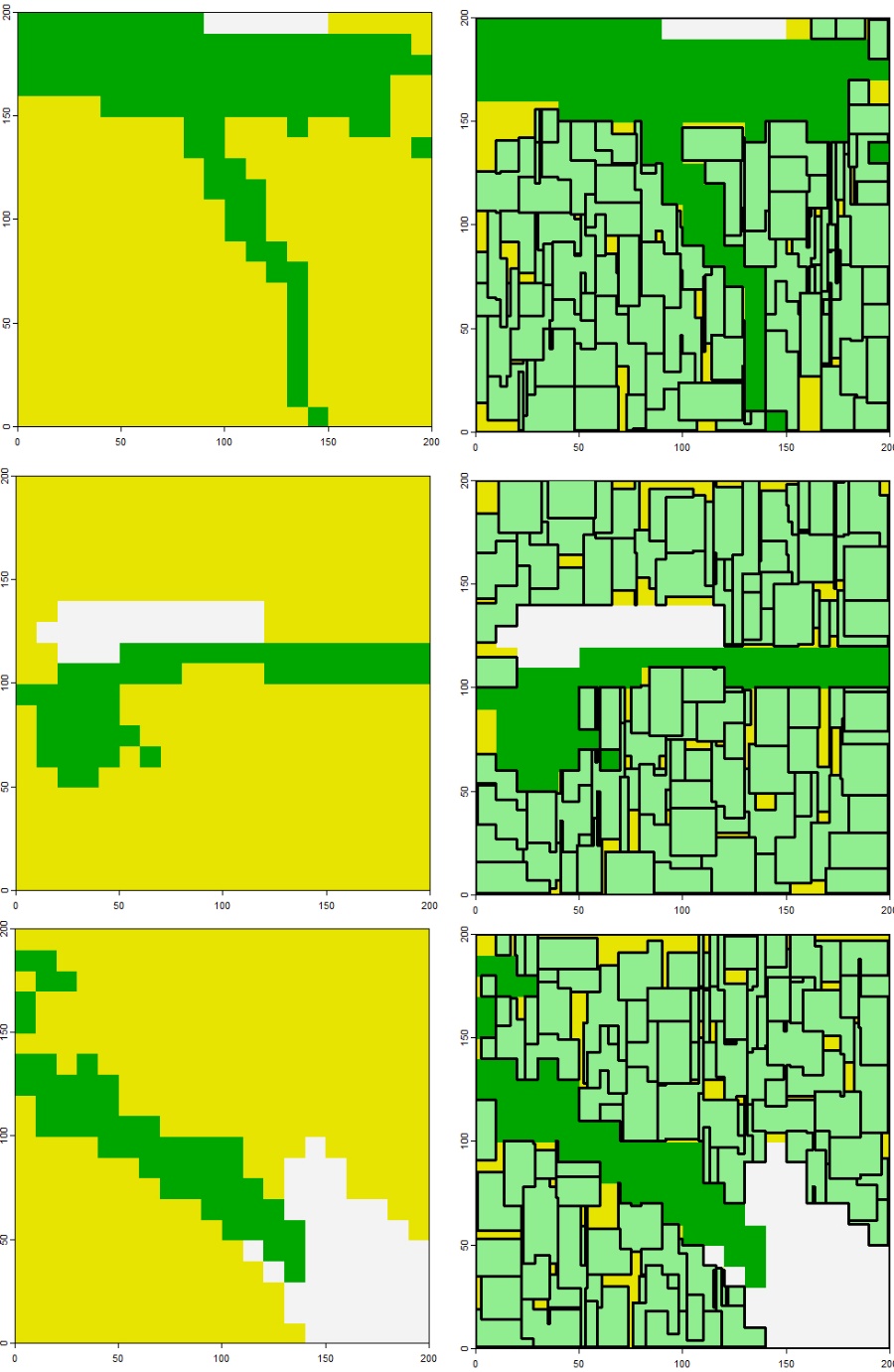


Fig S14: C) example of field placements on the ‘arable’ land use category of the Copernicus input map, and the ALGR function ‘*establish_pac’*

**Example 5: Monte Carlo uncertainty propagation**

Monte Carlo uncertainty propagation was used to assess how uncertainty in model inputs influences outcomes (Helton & Davis, 2003). Probability distributions were assigned to uncertain parameters, random samples were drawn, and the model was run repeatedly. The resulting ensemble of outputs was summarized to estimate overall uncertainty in predictions. We specified independent uniform priors for four generation parameters: *mean field size*, *sd field size*, *mean shape index*, and *sd shape index*. We drew N = 300 random samples, ran *ALGR* for each parameter set, and computed the number of fields and the mean field size, as well as core landscape metrics: contagion index (*contag*), *LPI*, and edge density (*ED*). We summarize uncertainty with the sample *mean*, *SD*, and 95% intervals (2.5th–97.5th percentiles). This process we repeated across a shift of up to +/- 40 cells in the value of the *mean* field size prior in order to demonstrate how the uncertainty shifts as the priors change. For the code of this example see notebook4.


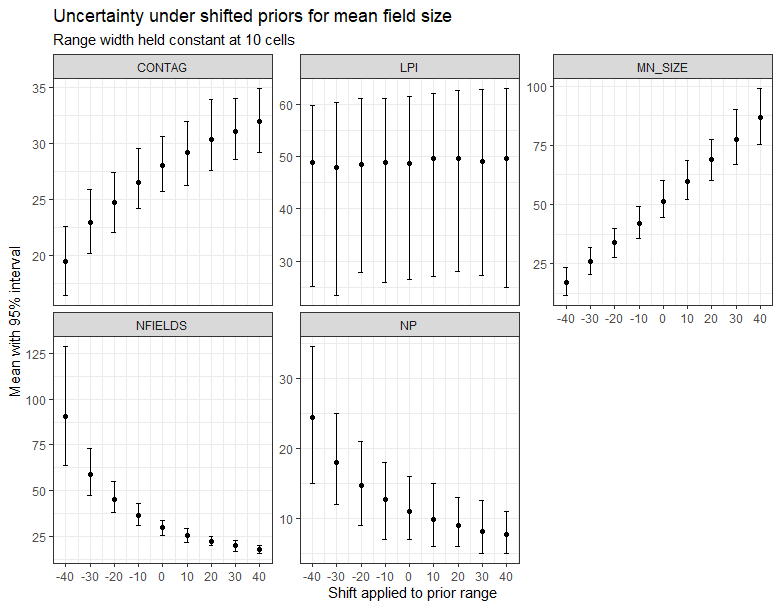


Figure S15: measuring uncertainty for different priors with a percentile range of 95%. For each prior value we ran a Monte Carlo simulation with N = 300 samples.

**References:**

Cong, R. G., Hedlund, K., Andersson, H., & Brady, M. (2014). Managing soil natural capital: An effective strategy for mitigating future agricultural risks? *Agricultural Systems*, *129*, 30–39. https://doi.org/10.1016/j.agsy.2014.05.003

Hanski, I. (1998). Metapopulation dynamics. *Nature*, *396*(6706), 41–49. https://doi.org/10.1038/23876

Helton, J. C., & Davis, F. J. (2003). Latin hypercube sampling and the propagation of uncertainty in analyses of complex systems. *Reliability Engineering & System Safety*, *81*(1), 23–69. https://doi.org/10.1016/S0951-8320(03)00058-9

Hesselbarth, M. H. K., Sciaini, M., With, K. A., Wiegand, K., & Nowosad, J. (2019). landscapemetrics: an open-source R tool to calculate landscape metrics. *Ecography*, *42*(10), 1648–1657. https://doi.org/10.1111/ecog.04617

Kaim, A., Cord, A. F., & Volk, M. (2018). A review of multi-criteria optimization techniques for agricultural land use allocation. *Environmental Modelling & Software*, *105*, 79–93. https://doi.org/10.1016/j.envsoft.2018.03.031

Tscharntke, T., Klein, A. M., Kruess, A., Steffan‐Dewenter, I., & Thies, C. (2005). Landscape perspectives on agricultural intensification and biodiversity – ecosystem service management. *Ecology Letters*, *8*(8), 857–874. https://doi.org/10.1111/j.1461-0248.2005.00782.x

Tscharntke, T., Tylianakis, J. M., Rand, T. A., Didham, R. K., Fahrig, L., Batáry, P., Bengtsson, J., Clough, Y., Crist, T. O., Dormann, C. F., Ewers, R. M., Fründ, J., Holt, R. D., Holzschuh, A., Klein, A. M., Kleijn, D., Kremen, C., Landis, D. A., Laurance, W., … Westphal, C. (2012). Landscape moderation of biodiversity patterns and processes - eight hypotheses. *Biological Reviews*, *87*(3), 661–685. https://doi.org/10.1111/j.1469-185X.2011.00216.x
